# Manganese-pyrochloric acid photosensitizer nanocomplexes against osteosarcoma: achieving both high activatability and high effectiveness

**DOI:** 10.3389/fbioe.2024.1485549

**Published:** 2025-02-11

**Authors:** Xuran Zhang, Jian Wang, Qun Feng, Li Lei, Zhiyong Zhu

**Affiliations:** ^1^ Department of Orthopedics, Fuxin Center Hospital, Fuxin, China; ^2^ Department of Sports Medicine and Joint Surgery, The People’s Hospital of Liaoning Province, Shenyang, China; ^3^ Department of Orthopedics, The First Affiliated Hospital of Jinzhou Medical University, Jinzhou, China; ^4^ Department of Pathology, Fuxin Center Hospital, Fuxin, China

**Keywords:** photodynamic therapy, nano-drug delivery systems, manganese-pyrochloric acid nanocomplexes, both high activatability and high effectiveness, osteosarcoma

## Abstract

**Introduction:**

The application of photodynamic therapy (PDT) is limited by unsatisfactory therapeutic efficacy and dose-dependent phototoxicity in clinical settings. Intravenous nano-drug delivery systems (NDDSs) hold promise for enhancing the delivery efficiency of photosensitive drugs, but often result in aggregation-caused quenching (ACQ) effects, preventing site-specific activation.

**Methods:**

We exploited manganese (Mn^2+^)–pyrochloric acid (PPa) nanocomplexes coordinated using the photosensitizer PPa and metal Mn ion for the treatment of osteosarcoma. The nanocomplexes were precisely co-assembled in water to stably co-deliver Mn^2+^ and PPa, enabling tumor-specific release and fluorescence recovery.

**Results:**

Following laser irradiation, the activated PPa significantly enhanced the killing effects on primary cancer cells. Additionally, Mn^2+^ ions activated the cyclic GMP-AMP synthase (cGAS)-stimulator of interferon genes (STING) pathway, promoting maturation of dendritic cells (DCs) and augmenting CD8^+^-mediated antitumor immune responses.

**Discussion:**

This study advances the on-demand activation of photosensitive drugs and photodynamic immunotherapy toward clinical applicability by exploiting Mn^2+^–PPa nanocomplexes with high activatability and effectiveness for targeted PDT and immunotherapy.

## Graphical Abstract

**Figure:**
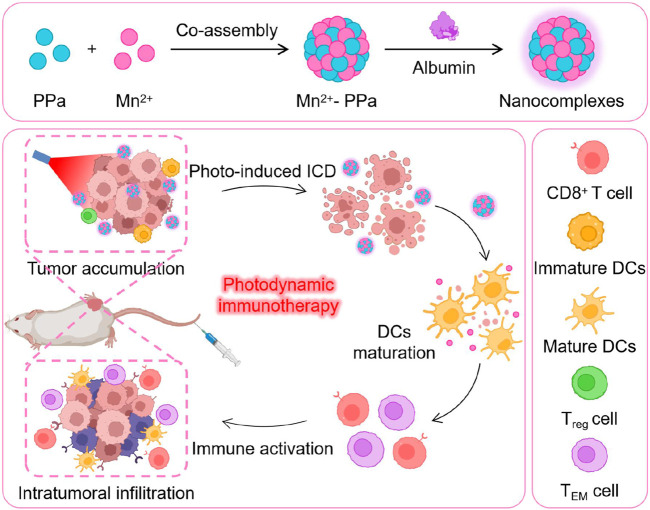

## 1 Introduction

Osteosarcoma is a highly malignant bone tumor that originates from osteoblasts. It can infiltrate the surrounding tissues and metastasize to other parts of the body, posing a serious health hazard (Li T. et al., 2023; Liang et al., 2023; Sapiano et al., 2023; Tian et al., 2023; Wang et al., 2023). It is the most common primary bone tumor in children and adolescents. Osteosarcoma is characterized by the presence of malignant mesenchymal cells that produce osteoid or immature bone (He et al., 2023; Liu et al., 2024; Luo et al., 2023; Wang W. et al., 2024; Wang Y. et al., 2024). Clinical manifestations of osteosarcoma typically include pain, swelling, local tenderness, and limited mobility in the affected area (Adine et al., 2024; Tai et al., 2024). Osteosarcoma necessitates prompt and aggressive treatment to improve outcomes and reduce the risk of complications (Wu et al., 2022; Zaaboub et al., 2022). The treatment of osteosarcoma typically involves a comprehensive approach. The primary treatment modalities include surgical resection of the tumor, adjuvant chemotherapy, and radiation therapy (Huang et al., 2022; Li et al., 2022). However, it is important to note that surgical resection may not be suitable for all patients, and chemotherapy and radiotherapy have limitations and potential side effects. Therefore, there is a pressing need for alternative treatment approaches to improve outcomes for patients with osteosarcoma (Yu et al., 2022).

Photodynamic therapy (PDT) is an emerging and promising modality for the treatment of osteosarcoma (Oskroba et al., 2024; Zhou et al., 2024). It relies on the use of photosensitizers, which, upon exposure to light, generate reactive oxygen species (ROS) that directly disrupt cellular structures and functions in lesion locations, resulting in the death of cancer cells. Currently, PDT has already found clinical applications in the treatment of superficial tumors, demonstrating its efficacy (Li G. et al., 2023; Ning et al., 2023). The effectiveness of PDT hinges on the key factor, which is the selection of photosensitizers. Different photosensitizers possess unique spectral characteristics, which directly influence the choice of appropriate light parameters. However, off-targeted photosensitizers result in dose-dependent phototoxicity and reduced therapeutic efficacy of PDT (Wang B. et al., 2024; Xu et al., 2023). Advancements in nanotechnology and extensive research on photosensitizers are expected to lead to the development of more precise and effective treatment strategies, offering patients improved therapeutic options. Despite their advantages, conventional nanocarrier-encapsulated photosensitizers suffer from low loading efficiency, poor stability, and aggregation-caused quenching (ACQ) effects, which significantly limit the efficiency of PDT (Ji et al., 2022; Zhang et al., 2022). Therefore, the development of novel nano-drug delivery systems (NDDSs) capable of highly efficient loading, targeted delivery, and on-demand release of photosensitizers at the tumor site is highly desirable for enhancing the effectiveness of PDT (Wei et al., 2022).

In this study, we elaborately constructed supramolecular nanocomplexes (Mn^2+^–PPa) by coordinating Mn^2+^ ions with photosensitizer pyrochloric acid (PPa). Mn^2+^ and PPa could be co-assembled in an aqueous medium with the addition of a small amount of albumin. The Mn^2+^–PPa nanocomplexes exhibited great colloidal stability under physiological conditions, favorable intratumoral accumulation, and rapid activation of PPa in the acidic tumor microenvironment. The PPa-produced ROS under laser irradiation suppressed primary osteosarcoma. In addition to their assembly stability and tumor-specific activation, Mn^2+^ ions can activate the stimulator of interferon genes (STING) pathway, facilitating the maturation of dendritic cells (DCs) and robust antitumor responses, which effectively inhibit the growth of distant osteosarcoma. Such a facile nanoplatform with both high activatability and high effectiveness could be poised to refine and expand the applications of PDT, potentially providing patients with enhanced treatment outcomes.

## 2 Results and discussion

### 2.1 Preparation and characterization of Mn^2+^–PPa nanocomplexes

**FIGURE 1:**
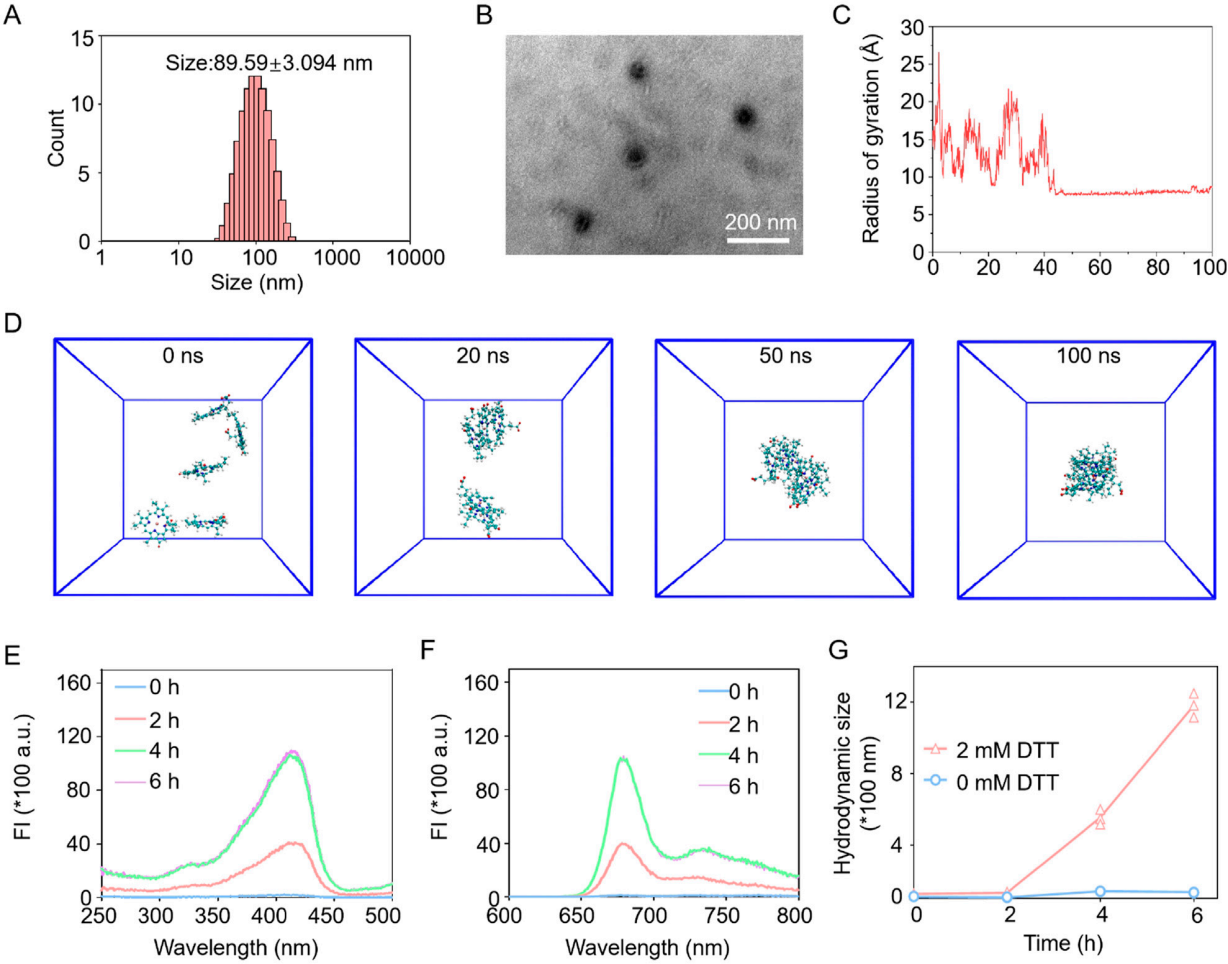
Characterization of Mn^2+^–PPa nanocomplexes. **(A,B)** Particle size distribution profiles and TEM images of nanocomplexes (scale bar = 200 nm); **(C)** RMSD of the interaction between Mn^2+^ and PPa; **(D)** Co-assembly progression of Mn^2+^–PPa nanocomplexes at different simulation times; **(E)** fluorescence excitation spectral changes in Mn^2+^–PPa nanocomplexes (2 μM, PPa equivalent) incubated in a medium with confirm concentrations of DTT at different time points (0, 2, 4, and 6 h); **(F)** fluorescence emission spectral change in Mn^2+^–PPa nanocomplexes (2 μM, PPa equivalent) incubated in a medium with confirm concentrations of DTT at different time points (0, 2, 4, and 6 h); and **(G)** particle size change in Mn^2+^–PPa nanocomplexes (2 μM, PPa equivalent) incubated in a medium with confirm concentrations of DTT at different time points (0, 2, 4, and 6 h).

Mn^2+^–PPa nanocomplexes were built by one nanoprecipitation method. In this study, we demonstrated that Mn^2+^ could serve as an assembly promoter to drive PPa to assemble into uniform nanostructures. To potentiate the stability of nanocomplexes, albumin was chosen as a stabilizer to contain hydrophilic and spherical nanostructures with a particle size of approximately 90 nm (Figures 1A,B; Supplementary Figure S1). Molecular dynamics (MD) simulation studies were conducted to investigate the formation mechanisms of nanocomplexes. As presented in Figures 1C,D, the simulation system tended to stabilize over 50 ns. While the simulation system was stabilized, one single large Mn^2+^–PPa assembly emerged at 100 ns. The snapshots from MD simulations showed the assembly process, transitioning from the original disordered state to the final stable assembly at the molecular level. The results suggested that manganese ions played a crucial role in facilitating the coordination formation and maintaining the stability of Mn^2+^–PPa complexes.

Photosensitizers encapsulated in the conventional NDDSs result in ACQ effect-mediated fluorescence quenching. For Mn^2+^–PPa nanocomplexes, PPa from nanocomplexes would be activated and released by the tumor-reductive environment, leading to fluorescence recovery. In this section, the fluorescence spectra of nanocomplexes were evaluated upon the addition of dithiothreitol (DTT, a reductive GSH simulant) at an excitation wavelength of 415 nm and emission wavelength of 675 nm at different times. As presented in Figures 1E,F, the excitation fluorescence intensity of nanocomplexes was evidently potentiated when incubated with DTT in a time-dependent manner. The enhanced fluorescence intensity could be attributed to the activation of nanocomplexes, following the release of PPa triggered by DTT. Similar trends were observed in the emission fluorescence spectra. Moreover, the large hydrodynamic size of nanocomplexes observed upon incubation with DTT could be attributed to the alleviated ACQ effect, followed by the collapse of nanostructures induced by H_2_O_2_ (Figure 1G). These results greatly demonstrated that metal ion-mediated photosensitizer (Mn^2+^–PPa) nanocomplexes could serve as an effective approach to solve the dilemma from ACQ effects. Next, we investigated the stability of the nanocomplexes directly in serum, as shown in Supplementary Figure S2. The particle size did not change significantly within 24 h, demonstrating that the nanoparticle formulation has a certain degree of stability in serum. In order to investigate the release efficiency of nanocomplexes, we placed them in different concentrations of GSH (Supplementary Figure S3). The release efficiency of nanomaterials in high concentrations of GSH was much higher than that in low concentrations of GSH, indicating that nanomaterials have good release efficiency in the tumor microenvironment.

### 2.2 *In vitro* antitumor evaluation of Mn^2+^–PPa nanocomplexes

**FIGURE 2:**
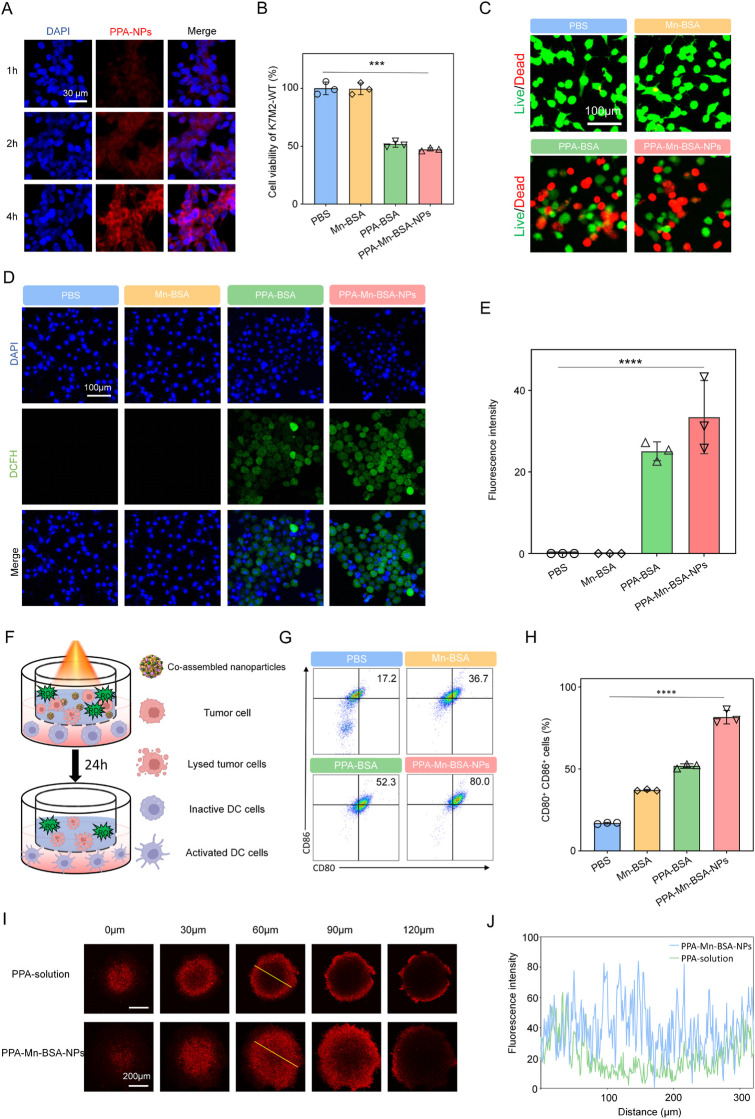
*In vitro* antitumor evaluation of Mn^2+^–PPa nanocomplexes. **(A)** Cellular uptake of Mn^2+^–PPa nanocomplexes at 1, 2, and 4 h through CLSM; cell viability changes **(B)** and images of LIVE/DEAD of K7M2 **(C)** staining assays upon Mn^2+^–PPa nanocomplex treatment. The mouse osteosarcoma cell line K7M2 was incubated with nanocomplexes for 24 h; **(D, E)** cellular ROS production in K7M2 cells stained with DCFH-DA after incubation with Mn^2+^–PPa nanocomplexes for 4 h under laser irradiation; **(F)** schematic diagram of the establishment of the DC maturation model. Mn^2+^–PPa nanocomplexes were co-cultured and treated with K7M2 cells under laser irradiation. Inactive DC cells were placed in the sublayer of transwells; **(G, H)** DCs from the model were collected for flow cytometry analysis. Gating and quantitative results for flow cytometry analysis of DC cells (CD11^+^CD80^+^CD86^+^). Data are presented as the mean ± SD (*n* = 3); Z-stack images **(I)** and quantitative results **(J)** for the penetration of Mn^2+^–PPa nanocomplexes in three-dimensional (3D) tumor spheroids. Data are presented as the mean ± SD (n = 5). ns, no significance. *p* > 0.05, *p < 0.05, **p < 0.01, ***p < 0.001, and ****p < 0.0001.

We first evaluated the cellular uptake of Mn^2+^–PPa nanocomplexes for 1, 2, and 4 h with K7M2-WT cells. The fluorescence intensity of PPa from nanocomplexes in cancer cells was found through confocal laser scanning microscopy (CLSM). As shown in Figure 2A, the nanocomplexes exhibited a time-dependent elevation in intracellular uptake, and higher intracellular contents of nanocomplexes were found. The cytotoxicity of nanocomplexes was next assessed using the MTT assay (Figure 2B). Following laser irradiation, the nanocomplexes and PPa solution showed strong *in vitro* anticancer effects, while manganese ions had minimal cytotoxicity on 143B cells. These results were consistent with those of live/dead staining (Figure 2C). We then investigated the intracellular ROS generation under laser irradiation using a DCFH-DA staining approach, both qualitatively and quantitatively, following the previous instructions. As shown in Figures 2D,E, strong intracellular fluorescence intensities were simultaneously observed in the nanocomplexes and PPa solution, suggesting the activated release of PPa from nanocomplexes and alleviated ACQ effects. These findings confirmed the efficient cellular uptake of nanocomplexes and on-demand activation of PPa, which resulted in excellent *in vitro* anticancer activity.

It is well known that the immunostimulatory Mn^2+^ ion has DC-activating capacities. We investigated the activation effects of nanocomplexes on DCs in the following transwell plate experiments (Figure 2F). Flow cytometric analysis demonstrated that the laser irradiation-triggered disintegration of nanocomplexes promoted the expression of co-stimulatory molecules CD80 and CD86, enhancing the maturation ability of DCs (Figures 2G,H). In addition, we also explored the *in vitro* tumor penetration of nanocomplexes after exposure to the laser irradiation using CLSM. It was observed that nanocomplexes can penetrate throughout the multicellular tumor spheroids and deliver to the interior under the laser irradiation (Figures 2I,J). Thus, favorable immunoactivating effects and *in vitro* tumor penetration ability make nanocomplexes potential candidates for further *in vivo* research.

### 2.3 *In vivo* biodistribution and antitumor investigation

**FIGURE 3:**
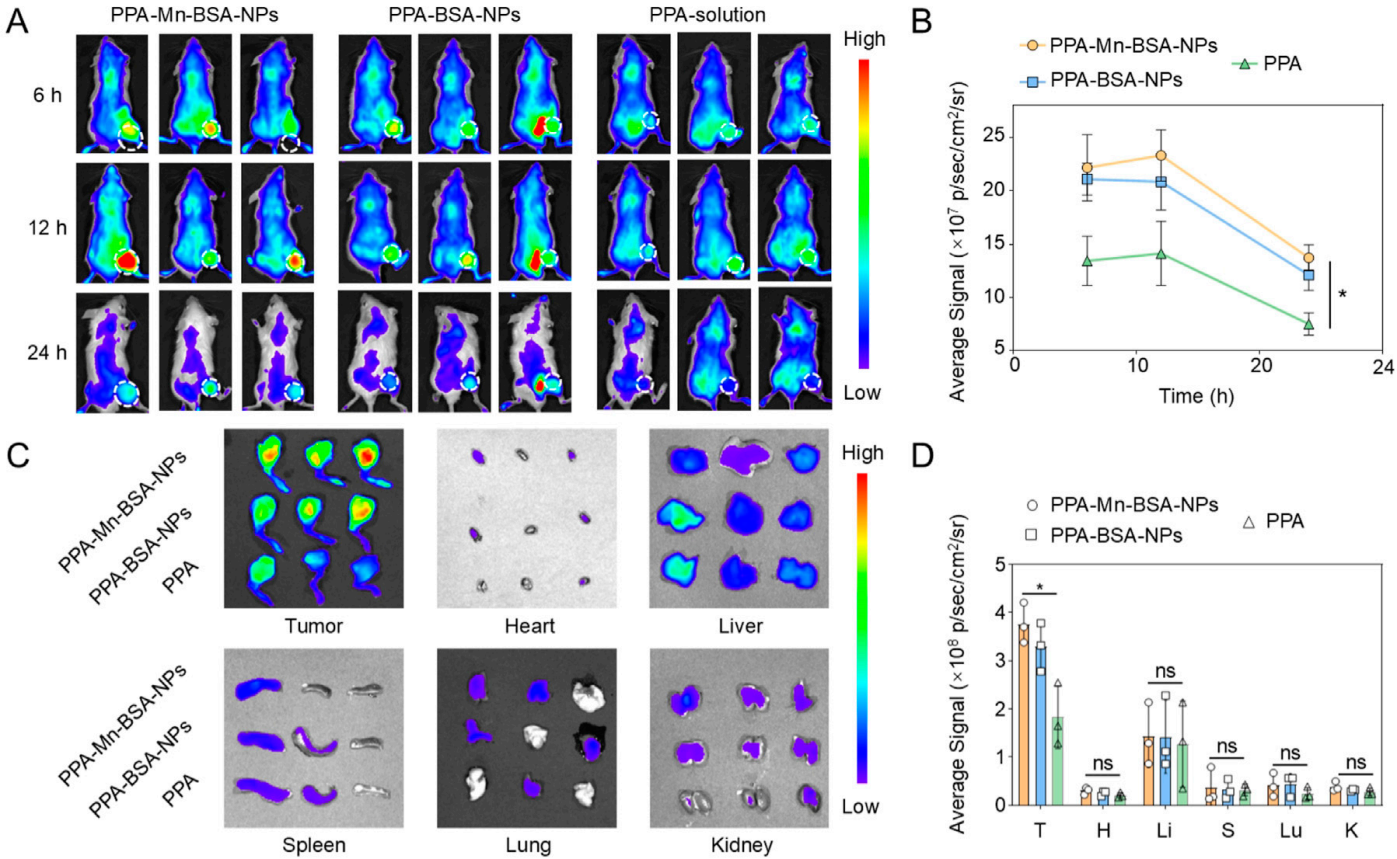
*In vivo* biodistribution and tumor accumulation. **(A)** Fluorescence images of PPA-Mn-BSA-NPs, PPA-BSA-NPs, and PPa solution distribution in mouse osteosarcoma cell line K7M2-bearing mice and *ex vivo* images of the main organs. **(B)** Quantification of PPA-Mn-BSA-NPs, PPA-BSA-NPs, and PPa solution in tumors after injection at various times (n = 3); **(C)** Distribution of PPA-Mn-BSA-NPs, PPA-BSA-NPs, and PPa solution in different organs after injection at 24 h (n = 3); **(D)** Quantification of PPA-Mn-BSA-NPs, PPA-BSA-NPs, and PPa solution in different organs after injection at 24 h (n = 3). Data are presented as the mean ± SD (n = 5). ns, no significance., *p* > 0.05, *p < 0.05, **p < 0.01, ***p < 0.001, and ****p < 0.0001.

The tumor-specific accumulation of drugs has a significant effect on the therapeutic outcomes of osteosarcoma. 143B osteosarcoma tumor-bearing BALB/c mice were used to evaluate the biodistribution of nanocomplexes, which could be observed by monitoring the fluorescence intensity of PPa in various organs and tumors. As shown in Figures 3A,B, PPa solutions were instantly eliminated from the body after intravenous injection, whereas nanocomplexes and PPA-BSA-NPs exhibited high intratumoral accumulation fluorescent intensity than PPa solutions within 24 h. After 24 h, we extracted multiple main organs (heart, liver, spleen, lung, and kidney) and tumor tissues for qualitative observation analysis and quantitative investigation, respectively. As shown in Figures 3C,D, nanocomplexes and PPA-BSA-NPs showed significant advantages over PPa solutions in terms of biodistribution, with higher intratumoral accumulation than in the main organs.

**FIGURE 4:**
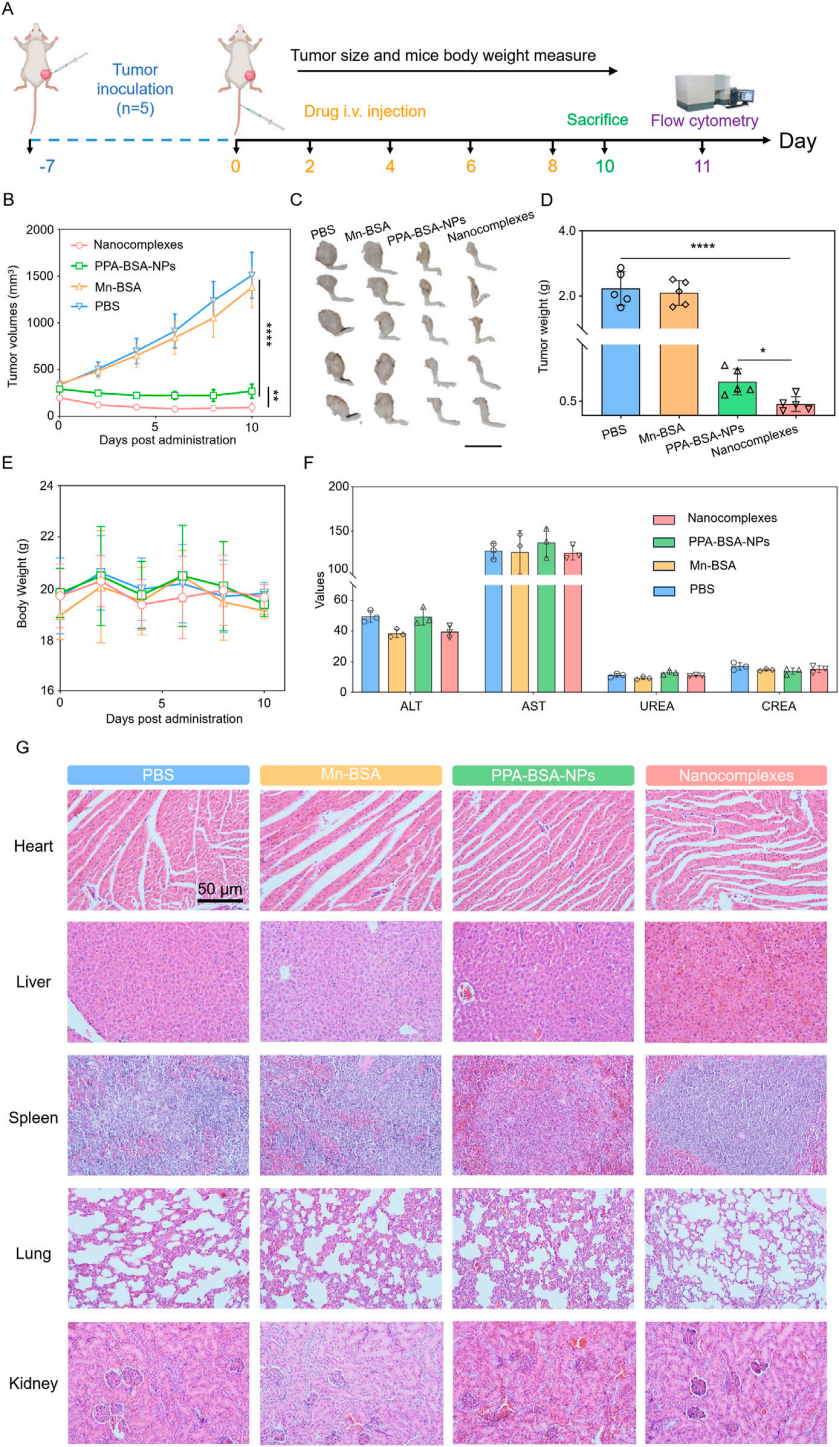
*In vivo* antitumor activity of Mn^2+^–PPa nanocomplexes against mouse osteosarcoma cell line K7M2 xenograft tumors. **(A)** Schematic illustration of treatment schedule; **(B)** tumor growth profiles treated with different formulations; images **(C)** and tumor weights **(D)** of osteosarcoma; **(E)** body weight changes; **(F)** hepatorenal function parameters. AST, aspartate aminotransferase (U L^−1^); ALT, alanine aminotransferase (U L^−1^); BUN, blood urea nitrogen (mmol L^−1^); CREA, creatinine (μmol L^−1^); and **(G)** H&E staining of various main organ slices after treatment with different formulations. Scale bars: 50 μm. Data are presented as the mean ± SD (n = 5). ns, no significance. *p* > 0.05, *p < 0.05, **p < 0.01, ***p < 0.001, and ****p < 0.0001.

Given great *in vitro* antitumor activity and *in vivo* biodistribution, *in vivo* antitumor efficacy of nanocomplexes was evaluated in BALB/c mice bearing primary 143B osteosarcoma tumors that were *i.v.* injected with PBS, Mn solution, PPa solution (irradiated with a 660 nm laser), and nanocomplexes (irradiated with a 660 nm laser), respectively (Figure 4A). As presented in Figure 4B, the tumor volume in the PBS group-/Mn solution group exhibited a rapid increase. The PPa solution group showed moderate tumor growth suppression, which was due to poor intratumoral accumulation characteristics (Figure 4C). The nanocomplexes showed superior tumor inhibitory capacity compared to the other groups. On Day 10, some of the mice were euthanized, and the tumor tissues were collected and weighed. The average tumor weight in the nanocomplex-treated group was the lowest, as shown in Figure 4D, agreement with tumor growth curve (Figure 4B). The survival rate of the treated mice demonstrated that the nanocomplexes had the strongest *in vivo* antitumor therapeutic efficiency. In addition, we also investigated the *in vivo* safety of various formulations during the treatment. A significant change in the body weight of mice was not found among all treated groups. After the treatment, the hematological parameters (aspartate aminotransferase (AST) and alanine aminotransferase (ALT) levels, blood urea nitrogen (BUN), and creatinine (CREA)) showed negligible indication of toxicity (Figure 4F). Furthermore, no evident histological changes were found in H&E-stained organs’ sections of the heart, liver, spleen, lung, and kidney (Figure 4G). These results revealed that nanocomplexes had a potent antitumor effect but no apparent toxic effect.

### 2.4 *In vivo* immunoactivation evaluation

**FIGURE 5:**
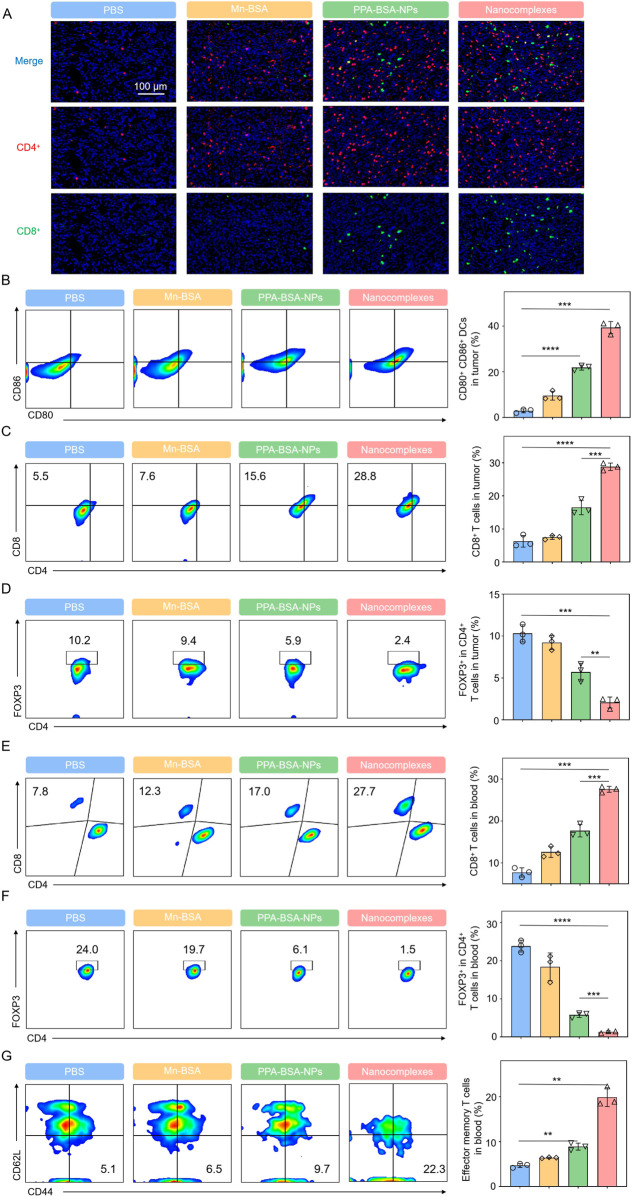
*In vivo* immunoactivation capacity of Mn^2+^–PPa nanocomplexes. **(A)** Immunofluorescence images of CD4^+^ and CD8^+^ T cells in tumors. Scale bars: 100 μm. **(B)** Representative flow cytometric evolution images and relative quantification of DC cells (CD45^+^CD11c^+^CD80^+^CD86^+^) in the tumor; **(C)** representative flow cytometric evolution images and relative quantification of CD8^+^ T cells (CD45^+^CD3^+^CD8^+^) in the tumor; **(D)** representative flow cytometric evolution images and relative quantification of Treg cells (CD45^+^CD3^+^CD4^+^FOXP3^+^) in the tumor; **(E)** representative flow cytometric evolution images and relative quantification of Treg cells (CD45^+^CD3^+^CD4^+^FOXP3^+^) in blood; **(F)** representative flow cytometric evolution images and relative quantification of Treg cells (CD45^+^CD3^+^CD4^+^FOXP3^+^) in blood; and **(G)** representative flow cytometric evolution images and relative quantification of effector memory T cells (CD45^+^CD3^+^CD8^+^CD62L^−^CD44^+^) in blood. Data are presented as the mean ± SD (*n* = 3). ns, no significance. *p* > 0.05, *p < 0.05, **p < 0.01, ***p < 0.001, and ****p < 0.0001.

To evaluate the mechanisms underlying the strong oncolytic effects triggered by nanocomplexes, immune responses in different treatment groups were evaluated. The mice were euthanized, and tumors and blood were extracted and collected after the last treatment for flow cytometric analysis. As presented in Figures 5B–D, Mn^2+^–PPa nanocomplex treatment could facilitate the highest level of intratumoral DC maturation compared to other groups. Furthermore, the Mn^2+^–PPa nanocomplex-treated group elevated the infiltration of CD8^+^ T cells and CD4^+^ T cells in the tumors, consistent with the observation shown in Figure 5A. In addition to the increased percentages of DCs and CD8^+^/CD4^+^ T cells, solid malignant tumors feature an immunosuppressive tumor microenvironment (TME) that has been regarded as one of the main factors of therapeutic resistance. The nanocomplex treatment contributed to evidently decreased regulatory T cells (Treg cells: CD45^+^CD3^+^CD4^+^FOXP3^+^ T cells) in the tumor, demonstrating that immunosuppressive conditions in the tumor were effectively alleviated. Moreover, the percentage of CD8^+^ in blood was upregulated, as shown in Figure 5E, while the immunosuppressive Treg cells in blood exhibited a remarkable reduction, as shown in Figure 5F, suggesting that the nanocomplexes could activate robust systemic immune responses. Interestingly, the proportion of memory T cells (CD45^+^CD3^+^CD8^+^CD62L^−^CD44^+^) in the nanocomplex-treated group was higher than in other groups (Figure 5G). This supramolecular nanocomplex treatment not only triggered the strongest immune effect but also induced immune memory protection.

## 3 Conclusion

To sum up, a supramolecular coordination strategy was used to successfully construct Mn^2+^–PPa nanocomplexes with both high activatability and high effectiveness for enhanced tumor treatment. The nanocomplexes could be significantly helpful for effectively overcoming the ACQ effect and greatly potentiating photodynamic therapeutic activity. Moreover, released Mn^2+^ promoted the maturation of DCs through the activation of the cGAS-STING signaling pathway, facilitating intratumoral infiltration of CD8^+^ T lymphocytes. Interestingly, nanocomplexes further boosted systemic antitumor immune memory responses. This novel coordination nanosystem offers an insight into the rational design of advanced PDT-based nano-DDS for highly efficient on-demand activation and amplified therapeutic performances.

## 4 Methods and materials

### 4.1 Materials

PPa was obtained from Ruixi Biotechnology Co. Ltd. (Xian, China). αLA, DTT, and N, N-dimethylformamide (DMF) were purchased from Aladdin Bio-Chem Technology Co., Ltd. (Shanghai, China). FBS was provided by Gibco, Invitrogen Corp., USA. ROS probe DCFH-DA, DAPI, Annexin V-FITC/PI Apoptosis Kit, Live–Dead Cell Staining Kit, penicillin–streptomycin, and Dulbecco’s modified Eagle’s medium (DMEM) were supplied by Dalian Meilun Biotechnology Co., Ltd. (Dalian, China). Hoechst 33342 and MTT were provided by Beijing Solarbio Technology Co., Ltd. (Beijing, China). Anti-CD 3, anti-CD 4, anti-CD 8, anti-Foxp3, anti-CD 80, and anti-CD 86 antibodies were obtained from BioLegend.

### 4.2 Animals and cells

The mouse osteosarcoma cell line K7M2 was acquired from COBIOER Biotechnology Co., Ltd. (Nanjing, China). The K7M2 cell line was incubated in DMEM with 10% FBS. The female BALB/c mice were supplied by Beijing HFK Bioscience Co. Ltd. (China). Animal experiments were performed according to the Guide for the Management and Use of Laboratory Animals and were authorized by the Institutional Animal Ethics Committee (IAEC) of Shenyang Pharmaceutical University.

### 4.3 Preparation of Mn^2+^–PPa nanocomplexes

Methanol was utilized as the solvent of PPa and MnCl_2_ (w:w, 1:10), and the complex solution was prepared at a concentration of 10 mg/mL. BSA was prepared into a solution of 10 mg/mL using ddH_2_O as the solvent. After that, 200 µL of the complex solution was added dropwise to a vial containing 1 mL of BSA-containing ddH_2_O and stirred using a magnetic stirrer for 10 min. The Mn^2+^–PPa nanocomplexes were formed with uniform particle size through a one-step nano-precipitation approach.

### 4.4 Characterization of Mn^2+^–PPa nanocomplexes

The size and zeta potential of Mn^2+^–PPa nanocomplexes were measured using a Zetasizer instrument (Malvern, United Kingdom). Then, the appearance of Mn^2+^–PPa nanocomplexes was observed with transmission electron microscopy (TEM, JEOL, Japan).

### 4.5 MD simulation

The composite structure of PPa and Mn^2+^ ions was drawn using MarvinSketch 24.1.0. Gaussian 09 was used to optimize the structure using the B3LYP functional and def2SVP basis set, and the single-point energy was calculated using the B3LYP functional and def2TZVP basis set. In Gaussian calculations, the SCRF implicit solvent model was used to simulate the water environment, and Mn radius was set to 2.0 Å. The RESP charge was calculated in Multiwfn based on the wave function computed by Gaussian, the GAFF2 force field parameters of the complex were generated by ACPYPE, and the parameters related to Mn^2+^ were computed and completed by Sobtop. PACKMOL was used to evenly place five PPa–Mn^2+^ complexes into a rectangular box with a side length of 50 Å, and the box was filled with TIP3P water molecules. A 100 ns molecular dynamics simulation was performed using GROMACS 2023 under the amber 14 force field, and the radius of gyration was calculated.

### 4.6 *In vitro* fluorescence recurrence assay

The Mn^2+^–PPa nanocomplexes were incubated with DTT, and the fluorescence spectra were detected at an excitation wavelength of 415 nm and emission wavelength of 675 nm under different time conditions. At the same time, the hydrodynamic size of the Mn^2+^–PPa nanocomplexes was measured with or without DTT.

### 4.7 Cellular uptake of Mn^2+^–PPa nanocomplexes

The K7M2 cells were inoculated in the six-well plate. When the cells reached approximately 80% confluence, the original medium was discarded, and the cells were washed twice with PBS. PBS solution containing Mn^2+^–PPa nanocomplexes (10 μg/mL, PPA equivalent) was added for 1 h, 2 h, and 4 h, after which the cells were observed via CLSM. DAPI was used to stain the K7M2 cells.

### 4.8 Cytotoxicity assay

K7M2 cells were cultured in 96-well plates to reach approximately 80% confluence. The cells were then treated with 150 μL of a fresh culture medium containing Mn-BSA, PPA-BSA, and nanocomplexes and cultured for 24 h under dark conditions. The PPA-BSA and Mn^2+^–PPa nanocomplex groups were exposed to laser light (660 nm 20 mW/cm^2^ 5 min) after 4 h of incubation. Then, the liquid in the 96-well plates was aspirated, and 150 μL of the MTT solution (1 mg/mL) was added to each well and placed in an incubator for 4 h. Finally, the MTT solution was replaced with dimethyl sulfoxide (100 μL), and the optical density value (490 nm) was recorded using a microplate reader.

In addition, the cytotoxicity of Mn^2+^–PPa nanocomplexes was evaluated using the Live–Dead Cell Staining Kit. K7M2 cells were seeded onto 12-well plates and treated as described above. After treatment, the cells were harvested, washed with PBS, stained with Annexin V-FITC/PI, and finally observed via CLSM.

### 4.9 ROS detection

The K7M2 cells were inoculated into a six-well plate. When the cells reached approximately 80% confluence, the original medium was discarded, and the cells were washed twice with PBS. The cells were incubated with DCFH-DA ROS probe for 1 h. They were then treated with PBS, Mn-BSA, PPA-BSA, and Mn^2+^–PPa nanocomplexes for 4 h, respectively. Subsequently, the liquid was discarded; the cells were washed with PBS, incubated with DIPA for 20 min, and finally observed via CLSM.

### 4.10 Evaluation of the immune activation ability

The K7M2 cells were cultured to the upper layer of the transwell plate, and the DC cells were cultured to the lower layer of the transwell plate. After overnight incubation, PBS, Mn-BSA, PPA-BSA, and Mn^2+^–PPa nanocomplexes were added for 24 h, respectively. Then, the cells were collected, labeled with antibodies, and subsequently measured and quantified by flow cytometry.

### 4.11 Tumor penetration of Mn^2+^–PPa nanocomplexes

We investigated *in vitro* tumor penetration of nanocomplexes in three-dimensional (3D) tumor spheroids. The K7M2 cells were cultured into 3D tumor spheroids. After that, the fresh culture solution was replaced regularly. When 3D tumor spheroids reached approximately 350 µm diameter, they were incubated with PPa solutions and Mn^2+^–PPa nanocomplexes for 4 h, respectively. Fluorescence was detected with Z-stack imaging by CLSM.

### 4.12 *In vivo* biodistribution

K7M2 osteosarcoma tumor-bearing BALB/c mice were used to evaluate the biodistribution of nanocomplexes. K7M2 tumors were inoculated into the tibia cavity of BALB/C mice to form osteosarcoma *in situ*. When the tumor volume reached 350 mm^3^, the tumor-bearing mice were injected with PPa solutions (n = 3) or Mn^2+^–PPa nanocomplexes (n = 3) intravenously. The images were captured at 6, 12, and 24 h after injection using an IIVIS Lumina Series III. The major organs and tumor-bearing legs were separated for *ex vivo* luminescence imaging after sacrifice.

### 4.13 *In vivo* antitumor effect

K7M2 osteosarcoma tumor-bearing BALB/c mice were used in this study. The mice were i.v. injected with PBS, Mn-BSA, PPa solution (irradiated with a 660 nm laser), and Mn^2+^–PPa nanocomplexes (irradiated with a 660 nm laser), respectively. Meanwhile, the changes in the tumor volume and body weight were recorded during treatment. The death of the mice was also recorded, and the survival curve was drawn.

### 4.14 *In vivo* toxicity of Mn^2+^–PPa nanocomplexes

After the experiment, the mice in each group were euthanized, and their main organs, including the heart, liver, spleen, lung, and kidney, were taken. Then, an H&E staining experiment was performed. At the same time, serum was collected, and AST, ALT, BUN, and CREA levels were analyzed.

### 4.15 *In vivo* immunoactivation evaluation

For tumor-infiltrated lymphocyte analysis, tumor samples were separated and digested using collagenase IV and hyaluronidase for 90 min to acquire single-cell suspensions. Then, cells were washed with RBC lysis buffer twice to remove red blood cells. The cells were counted and incubated with flow cytometric antibodies on ice for 30 min before testing. After that, the tumor cells were prepared as single-cell suspensions using a filter screen. CD8^+^T cells in tumor cell suspensions were stained with FITC-CD3, Percp-CD4, and APC-CD8a. Regulatory T cells (CD3^+^CD4^+^Foxp3^+^) in suspensions were stained with FITC-CD3, PerCp-CD4, and PE-Foxp3. Mature DCs (CD80^+^CD86^+^) were stained with APC-CD86 and FITC-CD80. The population of T cells, DC cells, and FOXP3^+^ cells in the tumor and the population of T cells, FOXP3^+^ cells, and memory T cells in blood were detected by flow cytometry.

### 4.16 Statistical analysis

Data are shown as the mean ± SD, and statistical significance was conducted using a two-tailed Student’s t-test (*p < 0.05, **p < 0.01, ***p < 0.001, and ****p < 0.0001; ns denotes not significant). All data were analyzed using GraphPad Prism 8 and Microsoft Excel software version 2019.

## Data Availability

The original contributions presented in the study are included in the article/Supplementary Material; further inquiries can be directed to the corresponding authors.
