# Supplementary material for: Manganese-pyrochloric acid photosensitizer nanocomplexes against osteosarcoma: achieving both high activatability and high effectiveness
Source: Front Bioeng Biotechnol. 2025 Feb 11;12:1485549. doi: 10.3389/fbioe.2024.1485549 (PMC11850124; doi:10.3389/fbioe.2024.1485549)
Supplement: Supplementary file 1 [file DataSheet1.pdf]

Supplementary Information for

Manganese-pyrochloric acid photosensitizer nanocomplexes against osteosarcoma: Achieving both high activatability and high effectiveness

Xuran Zhang, *et al.*

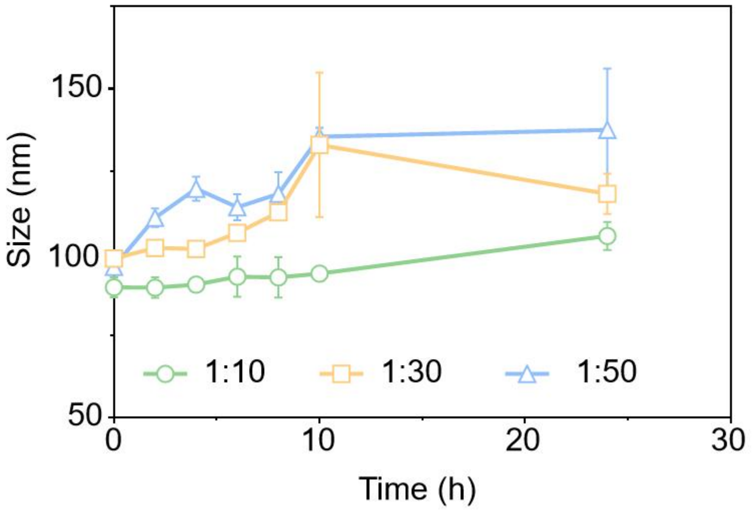

Supplementary Figure 1. Particle sizes distribution profiles at different mass ration of PPa and $MnCl_2$ (w:w, 1:10, 1:30, 1:50) at various times.

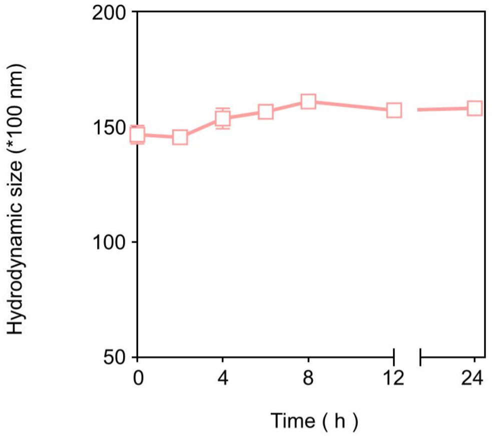

Supplementary Figure 2. Particle size changes of Nanocomplexes at different time points in 10% serum.

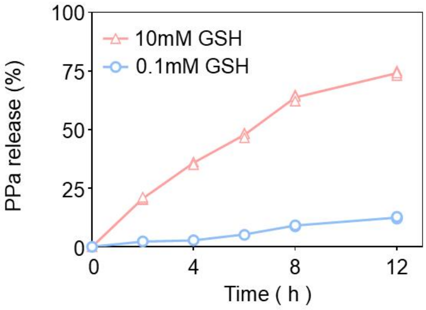

Supplementary Figure 3. PPA release rate of nanomaterials at different concentrations of GSH.
